# Late Glacial and Early Holocene human demographic responses to climatic and environmental change in Atlantic Iberia

**DOI:** 10.1098/rstb.2019.0724

**Published:** 2020-11-30

**Authors:** T. Rowan McLaughlin, Magdalena Gómez-Puche, João Cascalheira, Nuno Bicho, Javier Fernández-López de Pablo

**Affiliations:** 1 I.U. de Investigación en Arqueología y Patrimonio Histórico (INAPH), University of Alicante, Carr. de San Vicente del Raspeig, s/n, 03690 San Vicente del Raspeig, Alicante, Spain; 2 Interdisciplinary Center for Archaeology and Evolution of Human Behaviour (ICArEHB), Universidade do Algarve, Campus de Gambelas, 8005-139 Faro, Portugal

**Keywords:** palaeodemography, radiocarbon, palaeodiet, Palaeolithic, Mesolithic, Europe

## Abstract

Successive generations of hunter–gatherers of the Late Glacial and Early Holocene in Iberia had to contend with rapidly changing environments and climatic conditions. This constrained their economic resources and capacity for demographic growth. The Atlantic façade of Iberia was occupied throughout these times and witnessed very significant environmental transformations. Archaeology offers a perspective on how past human population ecologies changed in response to this scenario. Archaeological radiocarbon data are used here to reconstruct demographics of the region over the long term. We introduce various quantitative methods that allow us to develop palaeodemographic and spatio-temporal models of population growth and density, and compare our results to independent records of palaeoenvironmental and palaeodietary change, and growth rates derived from skeletal data. Our results demonstrate that late glacial population growth was stifled by the Younger Dryas stadial, but populations grew in size and density during the Early to Middle Holocene transition. This growth was fuelled in part by an increased dependence on marine and estuarine food sources, demonstrating how the environment was linked to demographic change via the resource base, and ultimately the carrying capacity of the environment.

This article is part of the theme issue ‘Cross-disciplinary approaches to prehistoric demography’.

## 1. Introduction

In this paper, we examine how human demography was influenced by past environmental change via changes in ecosystem productivity and carrying capacity. Recent palaeodemographic research for the Pleistocene–Holocene transition has revealed new insights into the dynamics of human populations, including different impacts of climate change, episodes of migration and population bottlenecks [1–4]. However, the reconstruction of population dynamics at a regional scale faces major empirical and methodological limitations when coastal regions are concerned. Factors of site preservation and visibility, and the complexities inherent to the calibration of marine samples with different local reservoir effects, have considerably limited the application of paleodemographic studies based on radiocarbon evidence. This problem is particularly relevant as coastal areas were especially sensitive to climatic and environmental changes, driving different foraging adaptations whose consequences in terms of population trajectories and settlement organization remains to be investigated.

The Atlantic façade of Iberia presents a paradigmatic case study. Located in the westernmost end of the Eurasian continent and the subject of significant changes in biological marine productivity driven by oceanic upwelling, previous research, e.g. [5–7], suggests that hunter–gatherer economy and culture comprise a mix of continuous and resilient adaptations, punctuated by rapid transformations associated with drastic rapid climatic events that would have affected the carrying capacity of the environment and driven a series of human adaptations.

**Figure 1.**
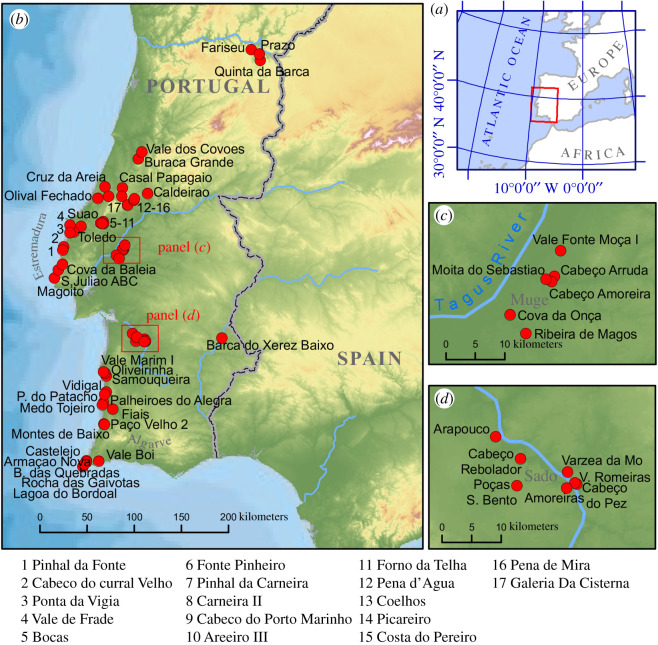
Maps indicating: (*a*) the location of the Atlantic façade of Iberia; (*b*) the distribution of post-LGM hunter–gatherer sites in the study region; and (*c,d*) the concentration of Late Mesolithic sites in the Muge and Sado estuaries, respectively. (Online version in colour.)

Following the Last Glacial Maximum (LGM), during the Magdalenian period (*ca* 20.5–13 kyr ago), foraging adaptations focused on terrestrial game, with increasing evidence of diversification [5,7]. The return of cool conditions in the Younger Dryas (YD) prompted a greater reliance on small prey, particularly lagomorphs, but culturally there was much continuity from earlier times, which remained during the following Epipaleolithic phase. The most dramatic shift in subsistence, settlement patterns and lithic technology occurred around 8200 cal. BP, with the widespread adoption of a repertoire of geometric microliths typical of the Late Mesolithic, e.g. [8,9]. It is during this phase that marine resources seem to become of paramount importance for the human population of the Atlantic façade of Iberia. The most important sites of this period are situated in palaeoestuaries that came under tidal influence at approximately the same time as their human exploitation began [8,10]. The Muge and Sado valleys in particular (figure 1) contain two concentrations of Mesolithic palaeoestuarine sites that consist of rich shell middens of intertidal species. These sites usually interpreted as semi-permanent settlement comprise thick shell-matrix with domestic waste enveloped within a complex stratigraphy [8]. They also contain a rich funerary record, with over 300 of human burials known.

Recently obtained ancient DNA evidence suggests there was a strong genetic continuity to these Final Pleistocene–Early Holocene changes in subsistence and material culture [3,11]. Therefore, the Atlantic region of Iberia offers a unique opportunity for studying long-term endogenous processes of demographic change in its ecological context from a multi-proxy perspective. This study aims to reconstruct long-term demographic dynamics using archaeological radiocarbon data. We introduce various quantitative methods that allow us to develop palaeodemographic and spatio-temporal models of population growth and density. Motivated by models of the relationship between ecosystem productivity and population size, e.g. [12], we also turn to a reconstruction of palaeodietary trends and draw comparisons with fertility rates derived from skeletal data.

## 2. Methods

We compiled a database of 62 archaeological sites and 371 radiocarbon dates, updating upon a synthesis previously published [4]. The data were subjected to two rounds of data screening, where dates with excessive error terms (greater than 200 years) were filtered from the database to reduce noise, as were dates where the archaeological association with human activity was not clear. Custom calibration was performed, using the IntCal20 marine and atmospheric curves [13], mixing these curves where necessary using published estimates of marine diet for each human bone sample. For both mixed-source and marine carbon reservoir samples, local offset values were used to calibrate the dates (electronic supplementary material, table S1).

Our population proxy is developed from summed probability densities (SPDs), calculated from the radiocarbon dates and normalized. To circumvent the likelihood that large numbers of dates from heavily sampled sites influenced the results, we used hierarchical cluster analysis [14] to reduce the dataset such that each site phase (defined for our purposes as the intergenerational period of 30 years) was represented by one radiocarbon date chosen at random from those available. The final number of dates retained was 284. Dates from open-air sites were summed separately to those from other sites, and their SPD was scaled according to the taphonomic correction proposed by Surovell *et al*. [15]. Dates from closed sites (caves and rockshelters) were then added to this SPD without any taphonomic correction. A confidence interval for the SPD was calculated using the bootstrapping method of [4].

Null hypotheses of exponential population growth were tested by fitting an exponential curve to the SPD and bootstrapping a confidence interval [4], and the periods of significant departure from this null model identified by comparing the bootstrapped confidence intervals of both curves. Adopting the rcarbon visual syntax, those areas of the bootstrapped SPD whose 95.4% confidence interval were above or below the confidence interval of 95.4% of the exponential null model were, respectively, shaded in blue and red, to denote statistically significant departures from the null model. We consider this approach is more robust against the calibration artefacts ‘false positives’ compared to methods that use a rolled mean SPD, e.g. [15,16]. Kernel density estimate (KDE) models for the data were calculated using the method of McLaughlin [17], also using one date per site phase, and this method was further developed to allow the calculation of dynamic models of the mean annualized growth rate. Unlike approaches that find the average geometric growth between fixed points in the SPD, such as century-wide ‘bins’ [16], we derived a fully continuous reading of growth from the radiocarbon data. This was done by numerically differentiating the bootstrapped KDE and expressing it as a time series with a defined confidence envelope. A similar analysis has been proposed by Brown [18], who pointed out that KDE-derived continuous growth models were a more useful exploratory tool than SPDs in this regard owing to their capacity for cancelling calibration noise.

To investigate the correlation between the SPD-based population proxy and other proxies, such as palaeotemperature proxies, we used Spearman's rank correlation coefficient, and subset our data to 500-year time slices stepped at 50-year intervals. The mean distance between sites was calculated by recording the pairwise Euclidean distance between each site [19] in 500-year time slices, using 50-year steps to develop these measurements into a timeseries.

For testing whether changes in population size are related to a widespread shift towards marine and estuarine food resources, we gathered together the details of all 54 published cases from the region where samples of ancient human tissue have been analysed for measurements of radiocarbon, stable carbon and stable nitrogen isotope ratios (electronic supplementary material). In this task, we were limited to a post-8500 cal. BP timeframe because to our knowledge, no earlier data are available from the region. We used locally estimated scatterplot smoothing (LOESS) regression to determine whether there was any time-dependent trend in the palaeodiet. This procedure used a Monte Carlo process to draw point samples from the calibrated radiocarbon ages, and used these to calculate 1000 individual LOESS models, each using a different set of randomly drawn ‘dates’. The fitted values for each of these 1000 predictions were averaged to generate a regression model of palaeodietary trends. Using this approach, the regression can be expressed with a confidence interval that conveys the uncertainty introduced by the radiocarbon calibration process as well as the uncertainty of the LOESS prediction.

The computer code (in R) we used to undertake all the analyses presented in this paper is included as the electronic supplementary material.

## 3. Results

**Figure 2.**
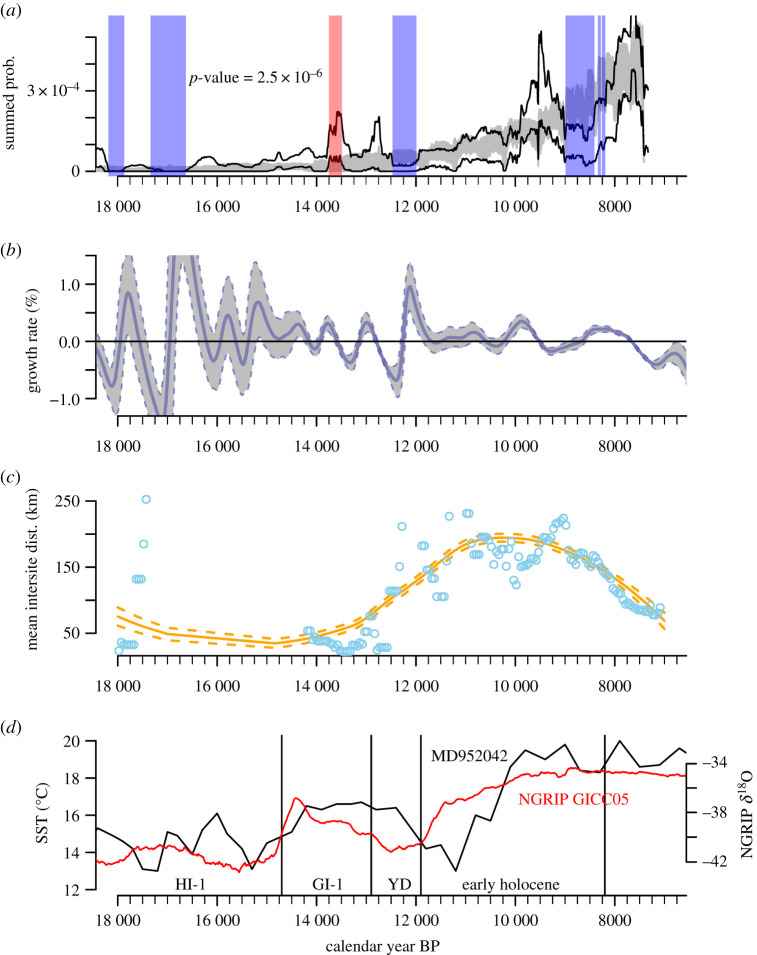
(*a*) The population proxy (the 95% confidence interval of 1000 bootstrapped simulations of SPDs of radiocarbon dates from archaeological contexts) compared to 1000 bootstrapped simulations of a fitted exponential null model (shaded area), with significantly high periods of activity heighted in red and low activity highlighted in blue. (*b*) Annualized dynamic growth rates (95% confidence interval) based on the first derivative of temporal Gaussian KDEs (150 year bandwidth) for radiocarbon dates from the Atlantic region. (*c*) Mean distance between all sites in 500-year time slices, stepped at 50-year intervals, demonstrating the concentration of sites before the Younger Dryas and a trend towards increased site density starting in the Early Holocene. (*d*) Sea surface temperature (SST) from the Iberian Atlantic coast [20] and a 400-year running mean of the oxygen isotope series from the NGRIP icecore using the GICC05 chronology [21,22]. (Online version in colour.)

Figure 2*a* shows the SPD population proxy fitted against a null exponential model of population growth for this timeseries [4,23]. Comparing the bootstraps' confidence envelopes, the probability that the two curves reflect the same underlying population dynamic is significantly low, *p* = 2.5 × 10^−6^. In figure 2*a*, we highlight cases where the outer bounds of the SPD confidence envelope depart from the null model. The SPD population proxy shows some significant negative departures from the exponential model, especially during climatic episodes of temperature downturn such as the Heinrich event 1 (HI-1, *ca* 18 000–15 000 cal. BP), the (YD *ca* 12 800–11 700 cal. BP) as well as the end of the Early Holocene. By contrast, we find a positive departure around 13 500, near the end of the Greenland Interstadial 1(GI-1 or Bølling/Allerød), before the YD commencement. The main trend is highly consistent with the histogram of site counts (see the electronic supplementary material, figure S1), indicating sporadic evidence for occupation until 14 500 cal. BP and oscillating activity thereafter, with the greatest density occurring in the Late Mesolithic. The Early Holocene section of the timeseries reveals that the increases in activity during the Mesolithic were abrupt, and the record is punctuated by downturns at 10 500 and 9000 cal. BP.

The comparison of the population proxy to high-resolution sea surface temperature records at regional level (figure 2*d*) shows how Final Pleistocene and Early Holocene climate changes impacted upon the human population at the Atlantic façade of Iberia. The YD is associated with a marked population decline across the Atlantic façade, which is consistent with a pattern also described at Iberian scale [4]. Proxy evidence, based upon marine core alkenone unsaturation ratios at a resolution of 300 years [20], indicates that the reduction in surface temperature of the Atlantic coast lagged behind the global situation and cold seawater conditions prevailed until a warming phase from 11 000 cal. BP to 10 000 cal. BP. This pattern coincides with the demographic trends derived from the radiocarbon data which show relatively low levels of human activity until the ocean waters warmed to the levels prevalent throughout the Holocene. The overall correlation between the population proxy and the Atlantic surface temperature is, therefore, quite high (Spearman's *ρ* 0.73), although this masks moments in time where there was an apparent causal relationship between the proxies but with a degree of lag. For example, population growth was interrupted around 10 500 cal. BP, just after a brief reversal in the ocean warming trend. Considering the correlation over 500-year windows stepped at 50-year intervals, an oscillating pattern of pronounced negative and positive relationships between the demographic and temperature proxies is observed (electronic supplementary material, Spearman's correlation; figure 2), broadly implying that there can be strong correlations between rising or falling temperatures and populations for millennia, but it takes some considerable time for this causal link to become established, presumably because of the lags introduced by the dynamics of the ecosystem, and the human sociocultural responses that take some number of generations to map on to demographic change.

The timing and relative strength of short-term fluctuations that caused the population proxy to temporarily deviate from this long-term pattern of growth are revealed by the dynamic growth model (figure 2*b*). The annualized growth rate can only be calculated with a usefully narrow degree of confidence for the periods that have a significant density of radiocarbon dates, which restricts our results to the Epipalaeolithic/Mesolithic. The dynamic growth rate model reveals two phases of Mesolithic growth that shared similar characteristics in terms of their growth rate and duration (electronic supplementary material, table S2). During the Early Mesolithic, we find a first phase of demographic growth starting at 10 300 ± 200 cal. BP lasting about 900 ± 500 years, with maximum growth rates of 0.40 ± 0.10% reached at 10 100 ± 300 cal. BP to reach a population maximum at 9400 ± 150 cal. BP. A second phase of population growth started about 8700 ± 100 cal. BP lasting about 950 ± 150 years. During this second phase, the dynamic model estimated a maximum growth rate of 0.25 ± 0.05%, at 8200 ± 100 cal. BP, during the onset of the Late Mesolithic period to reach maximum population at 7750 ± 50 cal. BP.

In order to investigate the spatial structure of the relative population changes, we conducted point-pattern analysis. Following recent approaches [24,25], we decided to do a simple first-order summary of average inter-site distance to determine whether changes in relative population levels were correlated with spatial clustering or dispersals of human populations. The average distances among all sites were calculated in 500-year time slices and graphically represented at 50-year intervals along a LOESS regression to summarize the main trends (figure 2*d*). The analysis shows the greatest clustering, with average inter-site distances less than 50 km, during the end of the Magdalenian period at GI-1. This concentration is located in the Portuguese Estremadura, where most of the empirical research on this period has been carried out [7]. With the onset of the YD, we find a sharp but sustained change in the average distances, reaching a fivefold increase at the beginning of the Early Holocene. Hence, lower SPD densities can be associated with a more dispersed population during times of dramatic environmental change. From *ca* 10 500 cal. BP onwards, we have identified a monotonic decreasing trend in the mean average distance between sites (from *ca* 200 to 100 km), a trend that culminated during the Late Mesolithic, when a clear clustering in a well-known settlement distribution associated with estuarine adaptations.

**Figure 3.**
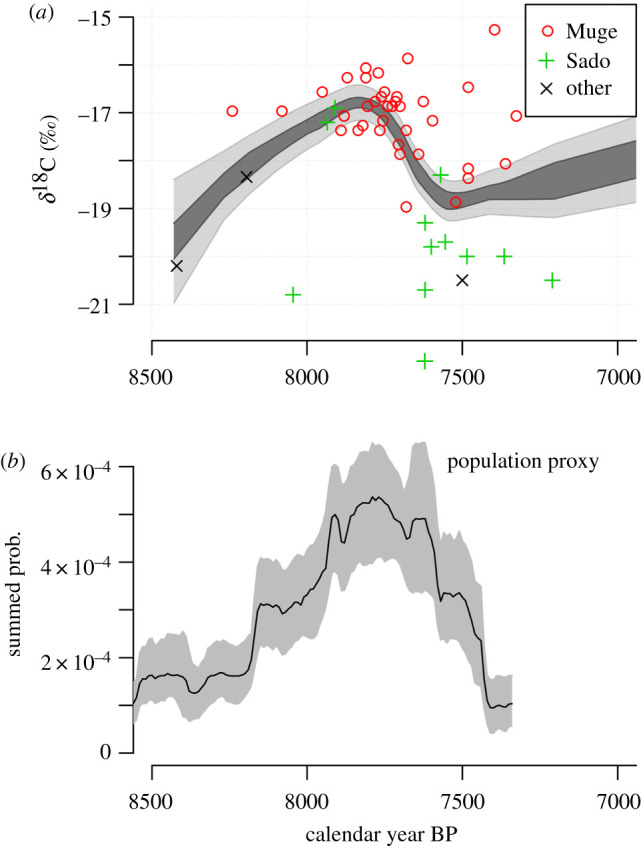
(*a*) Monte Carlo LOESS regression models for palaeodietary trends for the Later Mesolithic of the Atlantic façade of Iberia. More marine protein in the diet causes enriched (less negative) *δ*^13^C values. (*b*) For comparison, an archaeological radiocarbon SPD population proxy for the region zoomed to the same time window. Simultaneous peak in population and the use of marine resources occurred around 7800 cal. BP. (Online version in colour.)

Turning to the palaeodietary evidence from these densely settled estuaries (figure 3), our LOESS model indicates a trend of generally increasing reliance on marine food resources until 7800 cal. BP, and a shift back towards terrestrial resources from that point on. This matches the SPD palaeodemographic proxy, as peak Mesolithic activity is encountered at the point in time when marine resources such as fish, shellfish and marine mammals, were most prominent in the diet. The slight temporal difference between when burial was most intense at the Muge and Sado palaeoestuaries results in two clusters of results, showing that the Muge populations relied more on marine resources then the Sado group. Taken together with samples from elsewhere, the trend indicated in figure 3 emerges. Admittedly, this could be an effect of the different locations of the main estuaries and independent of dietary choice, although the pattern is weakly apparent even within the two main locations.

## 4. Discussion

In contrast with some studies [23,25], we have considered here a relatively small set of archaeological data, especially for the Late Glacial period. Given that we are interested in broad-scale trends in a relatively small region over a 10 000-year period, the density of our data compare favourably with other case studies (cf. [26]). In terms of the palaeodemographic modelling of our data, a likely complication is that sea-level rise reduced the visibility of earlier sites [7], resulting in an intense pressure of research on later, better-preserved sites. In adition, sedimentary processes have affected the preservation of archaeological contexts during the Middle Magdalenian *ca* 16–14.5 kyr ago, as pointed out by a recent study published after our paper was submitted [27]. New research undertaken in the context of commercial archaeology rescue operations in the Vouga Valley shows evidence of Magdalenian and Early Holocene radiocarbon dated sites further north Estremadura [27]. This suggests that the long term demographic patterns identified in this study for the Late Glacial might need to be revisited in the future, as new radiocarbon datasets are published. At face value, the radiocarbon data suggest that compared to other Iberian regions [4], the Late Glacial population of the Atlantic region was relatively low, but we cannot exclude the possibility that there were significant coastal settlements whose archaeological traces were lost to sea-level rise. It is not possible to completely eliminate such biases from our analysis, although we can deploy analytical tools that are sensitive to shorter-term changes, so that the dynamics within each phase are revealed without emphasizing the bias that results in absolute differences between phases. One such tool is the dynamic growth model (figure 2*b*), which expresses the instantaneous rate of change in the population without any influence from the absolute level. This approach is not without its drawbacks, as the uncertainty it expresses is large for poorly powered regions of time, but is presented here as a pragmatic solution to a difficult problem. Similarly, the timeseries of the mean site distance (figure 2*c*) is presented as a first-order abstraction of the archaeological record, and glosses over subtle but potentially important changes in the balance of clustering and dispersal, but is nonetheless useful for our purposes as a proxy of overall settlement density in the region. Although these potential pitfalls should be borne in mind, the Atlantic façade of Iberia in the post-LGM period offers an opportunity to study over the long-term how humans have adapted to rapid and significant environmental change. Our exploratory palaeodemographic and spatio-temporal models have added to the literature demonstrating that the archaeological record preserves signals of these density-dependent foraging adaptations [25]. The main implications of our findings are discussed below.

The period up to the commencement of the YD at 12 800 cal. BP can be characterized by exponential growth, albeit starting from a very small base during the LGM. From 15 000 cal. BP, the evidence suggests population growth that coincided with warm and relatively moist conditions and forest biome of the GI-1 interstadial. This environment sustained higher populations, but there is some evidence of short-term oscillations, with peaks in the SPD at 14 250, 13 500 and 12 750 cal. BP and low points in between. These fluctuations are also present, indeed more prominently so, in the record from elsewhere in Iberia [4] and this pattern can be seen as a representation of a wider trend towards larger populations in the Late Magdalenian enabled by optimum climatic conditions. For reasons not entirely clear, this activity was concentrated in the Estremadura in Portugal, west of the Tagus.

Population throughout Iberia was limited by cooler, dryer and generally unstable conditions of the YD. Despite adaptations to the new environment, reflected in the stone tools and animal bone assemblages from the archaeological sites in question [5], our radiocarbon evidence indicates a population level reduced from that of the preceding interstadial conditions. Climate can be evoked as the causal factor in this case; the surface temperature of the Atlantic at the Iberian coast lagged behind global temperature increases, assuming stable Holocene values at 10 000 cal. BP [20]. Pollen from marine sediments off the coast of Portugal indicates that at the beginning of the YD, deciduous vegetation decreases and evergreen oaks disappear, while *Artemisia* and *Ephedra*, species better adapted to steppe and semi-steppic environments increase [6,28]. Sea levels were rising rapidly, up to 1 m century^−1^, which would have rendered the coastline a transitory and rapidly changing environment. All these factors led to a situation whereby the small and dispersed population did not begin to grow until around 10 300 cal. BP principally because forest cover was lessened and the environment affected by unstable climate and weather conditions.

The SPD population proxy contains two marked phases of high population, both occurring in the Early Holocene. The first of these occurred from 10 300 cal. BP and lasted over a millennium. This activity was mainly concentrated in Estremadura west of the Tagus, with several sites in Algarve also known. The growth rate was approximately 0.3%, over twice the growth that occurred during the climatic amelioration of the GI-1 interstadial. The faunal record indicates that small land animal prey were still of primary importance, but marine resources featured more prominently in the diet too [5]. At 9400 cal. BP, this population entered a phase of decline, as did the human population elsewhere in Iberia [4]. However, at the Atlantic façade, the decline was of greater magnitude and lasted for longer; strong growth occurred in other regions around 8500 cal. BP, whereas in the Atlantic region, this expansion was more gradual in nature. This decline occurred as offshore sea temperatures reached their coldest values (figure 2*a*), so this localized worsening in environmental conditions could explain why this Iberian population lull was so pronounced at the Atlantic façade.

The period 9000–7500 cal. BP in Atlantic region saw steady growth and a dense concentration of sites. This boom in population occurred mainly in central-southern Portugal; in the Muge and Sado river estuaries, and also in a more dispersed pattern across Algarve. Estremadura also saw continuous occupation throughout this period, but at a lower level and with no additional growth from the levels already attained since the Early Holocene. Shell middens, which dominate the SPD signal at this point, provide rich archives of palaeoeconomic data. As such, the estuarine sites of Late Mesolithic Portugal have been very well studied and, thus, we can compare our radiocarbon-derived population models with other independent population proxies. Based on the proportion of juveniles in the burials, existing estimates of growth at Muge sites are high, around 0.6% per annum (although significantly less than the 1% growth for the Early Neolithic), driven by increasing fertility [29]. The dynamic growth model derived from the radiocarbon data for the Atlantic region as a whole suggests the regional growth was less than this, 0.27 ± 0.05% annum^−1^ which is much higher than usual for hunter–gatherer societies, thus highly compatible with the short-term demographic estimates drawn from skeletal data and reinforces the validity of our results.

In terms of its bioclimatic context, the Atlantic façade of the Iberian Peninsula presents an important intersection of ocean-driven weather prevailing over a Mediterranean forest biome. The global changes in climate that occurred since the Late Glacial period, therefore, express themselves uniquely in this landscape, and as such, human adaptations to the changing conditions can be expected to follow different trajectories than those elsewhere. In comparison with other Iberian regions, Lower Magdalenian populations seemed low and there is no signal of growth until 16 000 cal. BP, when interstadial conditions became established. Short- and medium-term instability in these conditions seems to have been a factor that limited growth (see the electronic supplementary material, Spearman's *ρ*). It is significant that when a notable phase of population growth occurred at 14 000 cal. BP, sea surface temperatures had stabilized and would remain so for two millennia. This was a watershed moment in the population history of the region because the colder and unstable conditions that ensued during this period eventually had the effect of reducing the population across Iberia, including in the Atlantic region. By contrast, when Holocene conditions were established, the relative density of population in the Atlantic façade was similar to or even higher than elsewhere in Iberia [4].

What enabled this growth? From an archaeological perspective, the record is one of continuity; trends in stone tool manufacture, settlement type and prey choice can be traced from the LGM until the start of the Late Mesolithic [5,30]. In earlier work, Bicho *et al.* [8] argued that the emergence of inland estuarine shell middens forming large shell mounds with persistent funerary activity areas resulted from a punctuated environmental change induced by the 8.2 kyr ago event, which caused the arrival of salt water in the Tagus palaeoestuary. Under this view, the Portuguese estuaries became foci of settlement because they provided resource-rich stable environments, and ones that were newly formed, unlike the coastal ecosystems that were adversely affected by the 8.2 kyr ago cold event. Earlier work has also suggested that evolving material culture in the region can be read as evidence for ongoing adaptations to a changing environment without significant population turnovers [5]. This is a view supported, or rather not contradicted, by the recent ancient genomic analysis of an individual from Moita do Sebastião, who shares a significant amount of ancestry with people who lived in Iberia since at least 19 000 cal. BP [3]. The radiocarbon evidence we consider here suggests this line of continuity was nonetheless unstable in terms of the population size. The data also illustrate how the formation of new maritime environments after the 8.2 kyr ago event either transformed the local fertility (as suggested by the bioarchaeological evidence) or permitted inward migration at an unprecedented rate. Resource availability played a role in enabling this growth, and our palaeodietary model (figure 3) suggests the gradual adoption of marine resources was key. Important too was a longer-term trend towards greater settlement density that can be traced over the preceding three millennia (figure 2*d*), and probably had a direct impact on the regional social organization. Crucially, all this occurred *before* the colonization of the region by Neolithic incomers. Elsewhere on the Atlantic façade, there are numerous other examples of intensification in the use of marine and aquatic resources during the closing centuries of the Mesolithic period. These include northern Iberia [31], Brittany [32], northern Britain [33] and Scandinavia [34], all of which saw intensive occupation and the development of shell middens before the introduction of agriculture. This raises the question of whether the Neolithic economy, rapidly expanding across Europe at this time [35], was influencing these fringes via edge effects. In our case study, it seems the Iberian intensification in the use of marine and estuarine resources could have been an endogenous, density-dependent process, although the mechanism of such external pressures remains to be formally modelled.

The recurring theme in this analysis has been the interplay between environmental instability and the resulting effects on human population levels. The YD in particular was constraining, but Late Glacial phases of warmer and less arid conditions correlate with increases to the population. The relative stability of Holocene conditions allowed people to move into closer proximity with one another, lowering the cost of forming kinship networks and ultimately enabling more children to be born. Such patterns of behaviour can be detected in many other prehistoric settings and the social consequences of increased prehistoric settlement density could have been profound.

## Supplementary Material

Electronic Supplementary Materials

## Data accessibility

All the data analysed and the R code designed for this study are supplied as the electronic supplementary material in an external repository: http://doi.org/10.5281/zenodo.3626791.
