# Supplementary material for: Late Glacial and Early Holocene human demographic responses to climatic and environmental change in Atlantic Iberia
Source: Philos Trans R Soc Lond B Biol Sci. 2020 Nov 30;376(1816):20190724. doi: 10.1098/rstb.2019.0724 (PMC7741096; doi:10.1098/rstb.2019.0724)
Supplement: Electronic Supplementary Materials [file rstb20190724supp1.docx]

**Electronic Supplementary Material**

**Late Glacial and Early Holocene human demographic responses to climatic and environmental change in Atlantic Iberia**

**T. Rowan McLaughlin^1^, Magdalena Gómez-Puche^1^, João Cascalheira^2^, Nuno Bicho^2^, Javier Fernández-López de Pablo ^1^***

*1*. *I.U. de Investigación en Arqueología y Patrimonio Histórico (INAPH), University of Alicante, Carr. de San Vicente del Raspeig, s/n, 03690 San Vicente del Raspeig, Alicante, Spain*

*2. Interdisciplinary Center for Archaeology and Evolution of Human Behaviour (ICArEHB), University of Algarve, Campus de Gambelas 8005-139 Faro, Portugal*

**Introduction**

The following supplementary information provides i) details about the data sources and calibration procedures; ii) additional results on the temporal frequency distribution of sites and radiocarbon dates of Atlantic Iberia; iii) complementary results on the Spearman’s rank correlation between paleoclimate and the SPD population proxy; and iv) summary statistics about the time series of mean inter-site distances.

## **Data sources of radiocarbon calibration**

For both mixed-source and marine carbon reservoir samples, local offset values were used to calibrate the dates (Table 1).

| ∆R value | Subregion | Sites | References |
| --- | --- | --- | --- |
| 69 ± 17 | Algarve | Vale Boi | Monge-Soares *et al*., 2016 |
| -110 ± 40 | Western Algarve | Castelejo | Valente *et al.,* 2014 |
| -116 ± 44 | Western Algarve | Barranco das Quebradas, Rocha das Gaivotas | Valente *et al.,* 2014 |
| 380 ± 30 | Central Portugal | Bocas, Forno da Telha | Bicho et al., 2010 |
| 95 ±15 | Estremadura portuguesa, Alentejo | Buraca Grande, Samouqueira I, Magoito, Casal Papagaio, São Julião, Paço Velho 2, Toledo, Pinhal da Fonte, Vale Frade, Fiais, Medo Tojeiro, Montes de Baixo, Armação Nova, Oliveirinha, Pedra do Patacho | Monge-Soares *et al.,* 2016 |
| 140 ± 40 | Muge shellmiddens | Cabeço da Arruda, Cabeço da Amoreira, Moita do Sebastião, Cova da Onça | Martins *et al*., 2008 |
| -150± 155 | Sado shellmiddens | Amoreiras, Arapouco, Cabeço do Pez, Várzea da Mó, Poças de São Bento, Vale Romeiras | Araujo, 2015 |

Table 1. Local ∆R values specifically applied to each site

**Temporal frequency distributions of sites and radiocarbon dates in Atlantic Iberia**

## This section provides additional information about the particular properties of the radiocarbon record of Atlantic Iberia for conducting radiocarbon palaeodemographic research. At exploratory level, we show the temporal frequency distribution of dated sites and radiocarbon dates in figure 1. Fig.1 A, shows the number of sites divided in 500-year bins following the methodology published in Fernández-López de Pablo et al., 2019. This plot clearly illustrates how open-air sites become dominant during the Early Holocene regarding the final Pleistocene and the Younger Dryas.

## Fig.1B illustrates the SPD against the temporal divisions of the regional archaeological sequence, showing pronounced peaks in the SPD signal at c. 9.5 kya and 8.7 kya due to the calibration process that require to be corrected. Finally, Fig.1.C shows the contribution of different sample types on the original SPD. Marine shell samples display an increasing contribution from c.12 kya to the end of the time series whereas animal and human bones become predominant during the Late Mesolithic (c.8.2 onwards).

Figure 1. Temporal frequency distribution of archaeological materials from Atlantic Iberia, visualised as (A) bar chart showing the number of sites with material dated to within 500-year bins, (B) SPD of archaeological radiocarbon dates from the region, with periodization adapted from Bicho and Haws (2012) and (C) SPD showing the composition of the radiocarbon dataset through time.

## **Spearman’s rank correlation between paleoclimate and the SPD population proxy**

## To investigate the correlation between the SPD-based population proxy and other proxies, such as palaeotemperature proxies, we used Spearman’s rank correlation coefficient, and subset our data to 500-year time slices stepped at 50-year intervals.

##

Figure 2. Correlation coefficient (Spearman’s rho) calculated for the Pailler and Bard (2002) Atlantic temperature proxy and the demographic proxy derived from the radiocarbon data, using 500-year windows running though the timeseries at 50 year intervals.

**Summary statistics about mean inter-site distances**

We calculated the mean distance between sites by recording the pairwise Euclidean distance between each site in 500-year time slices, and used 50-year steps to develop these measurements into a timeseries. Table 2 provides more detail about the degree of clustering and dispersal by calculating summary statistics at different scales of analysis, by considering separately sites within and outside a radius of 35km.

The values for all sites in the study area are median distance of 3.3 km to the nearest site, median of 17 km to sites within 35 km, and 144 km to sites over 35 km.

| **Statistic** | **18,000 to 14,000** | **14,000 to 12,000** | **12,000 to 10,500** | **10,500 to 9500** | **9500 to 8500** | **8500 to 7500** |
| --- | --- | --- | --- | --- | --- | --- |
| Number of sites | 5 | 9 | 9 | 12 | 17 | 31 |
| Median distance nearest site | 32 km | 22 km | 19 km | 6.1 km | 7.5 km | 3.3 km |
| Median distance to sites <35 km | 32 km | 25 km | 25 km | 12 km | 19 km | 9 km |
| Median distance to sites > 35 km | 54 km | 181 km | 200 km | 196 km | 192 km | 130 km |

Table 2. Descriptive statistics for distance between sites according to different time slices expressed in cal BP years.

# **References**

Araújo A C. 2015 A few steps backwards… in search of the origins of the Late Mesolithic. In Bicho N, Detry C, Price T D, Cunha E (eds.) *Muge 150^th^: The 150^th^ anniversary of the discovery of Mesolithic shellmiddens*. Cambridge Scholar Publishing. Vol. 2, 1-12 pp.

Bicho N, Haws J. 2012 The Magdalenian in central and southern Portugal: Human ecology at the end of the Pleistocene. *Quat. Int.* **272**–**273**, 6–16.

Bicho N, Umbelino C, Detry C, Pereira T. 2010 The emergence of Muge Mesolithic shell middens in Central Portugal and the 8200 cal yr BP Cold Event. *Journal of Island and Coastal Archaeology* **5**, 86-104.

Fernández-López de Pablo J, Gutiérrez-Roig M, Gómez-Puche M, McLaughlin R, Silva F, Lozano S. 2019 Palaeodemographic modelling supports a population bottleneck during the Pleistocene-Holocene transition in Iberia. *Nat. Commun.* **10**, 1872. (doi:10.1038/s41467-019-09833-3).

Martins J, Carvalho A, Soares A. 2008 A calibraçao das datas de radiocarbono dos esqueletos humanos de Muge. *Promontoria*, **6**: 73-94.

Monge-Soares A M, Gutiérrez-Zugasti I, González-Morales M, Matos-Martins J M, Cuenca-Solana D, Bailey G. 2016 Marine radiocarbon reservoir effect in Late Pleistocene and Early Holocene coastal waters off Northern Iberia. *Radiocarbon* **58**, 869-883.

Pailler D, Bard E. 2002 High frequency palaeoceanographic changes during the past 140 000 yr recorded by the organic matter in sediments of the Iberian Margin. *Palaeogeogr. Palaeoclimatol. Palaeoecol.* **181**, 431–452. (doi:https://doi.org/10.1016/S0031-0182(01)00444-8)

Valente, M.J., Dean, R. & Carvalho, A.F. 2014. Shell Middens in Western Algarve (Southern Portugal) during the Mesolithic and Early Neolithic: Functionality, subsistence, and material culture. In Roksandic, M., Souza, S.M., Eggers, S. & Burchell, M. (eds.) *The Cultural Dynamics of Shell-Matrix Sites*. University of New Mexico Press, 75-90.

.
